# Real-World data on efficacy of L-glutamine in preventing sickle cell disease-related complications in pediatric and adult patients

**DOI:** 10.3389/fmed.2022.931925

**Published:** 2022-08-01

**Authors:** Narcisse Elenga, Gylna Loko, Maryse Etienne-Julan, Randa Al-Okka, Ahmad M. Adel, Mohamed A. Yassin

**Affiliations:** ^1^Centre Hospitalier de Cayenne, Cayenne, France; ^2^Centre de reference de la drepanocytose, CHU de la Guadeloupe, Pointe-à Pitre, Guadeloupe, France; ^3^Centre de reference de la drepanocytose, CH de Fort de France, Fort de France, France; ^4^Department of Pharmacy, NCCCR Pharmacy, Hamad Medical Corporation, Doha, Qatar; ^5^Medical Oncology Department—Hematology Section, National Centre for Cancer Care and Research, Hamad Medical Corporation, Doha, Qatar

**Keywords:** L-glutamine, sickle cell disease, clinical outcomes, hemolysis parameters, vaso-occlusive crisis

## Abstract

**Background:**

L-glutamine has been shown to play an important role in the regulation of oxidative stress which is one of the key contributors to the pathophysiology of sickle cell disease (SCD). In a Phase 3 clinical trial, L-glutamine demonstrated a significant reduction in SCD-related complications including vaso-occlusive crises (VOCs), hospitalizations, and acute chest syndrome (ACS) compared to placebo in patients with SCD.

**Objective:**

The primary objective was to confirm the efficacy of L-glutamine (Endari^®^) therapy in pediatric and adult patients with SCD at follow-up time points of 24, 48 and 72 weeks.

**Methods:**

In the observational study, nineteen patients with SCD were treated orally with L-glutamine twice daily for 72 weeks. Clinical and laboratory parameters were measured at baseline and follow-up time points. Patients with severe VOC and ACS were hospitalized. Blood transfusion was given in case of ACS and uncontrolled pain associated with VOC despite administration of the highest dose of intravenous (IV) narcotic.

**Results:**

Compared to baseline, patients had significantly fewer pain crises (median change from 3.0 to 0.0; *P* < 0.00001), hospitalizations (median change from 3.0 to 0.0; *P* < 0.00001), days of hospitalization (median change from 15.0 to 0.0; *P* < 0.00001), and blood transfusions (median change from 3.0 to 0.0; *P* < 0.00001) at 24, 48, and 72 weeks following L-glutamine therapy. Moreover, there was a drastic decrease in the number of ACS events during this time. A significant increase was observed in mean hemoglobin levels and hematocrit proportions from baseline to 72 weeks (*P* < 0.001). Conversely, compared to baseline, mean reticulocyte counts and lactate dehydrogenase (LDH) levels were considerably lower at follow-up time points (*P* = 0.003 and *P* < 0.001, respectively). No patient reported treatment-related adverse events.

**Conclusion:**

Although the sample size was small, our data clearly demonstrated that L-glutamine therapy was safe and significantly improved clinical outcomes and hemolysis parameters in patients with SCD.

12pt

## Introduction

Sickle cell disease (SCD) is a heterogeneous group of life-threatening inherited blood disorders that is prevalent worldwide and affects millions of people (1). An estimate by Piel et al. (2) predicted the overall number of births affected by SCD to be 14.2 million between 2010 and 2050 (2). SCD occurs due to the presence of abnormal hemoglobin S (HbS) which is formed by a point mutation resulting in a single amino acid substitution (glutamic acid to valine) at position 6 in the gene that encodes for the β-globin chain of hemoglobin (3, 4). Red blood cells (RBCs) undergo alteration in structure and function as a result of stress-induced intracellular polymerization of HbS (4). These deformed (“sickle shaped”) RBCs become very adhesive and upon interaction with white blood cells (WBCs) and the endothelium cause chronic hemolysis and occasional microvascular occlusion in multiple body organs (4). These processes can result in serious clinical complications that include acute pain (also called as vaso-occlusive crisis or VOC), tissue ischemia, multi-organ damage, stroke, acute chest syndrome (ACS) (5, 6).

The only approved curative treatment for SCD patients is bone marrow transplantation (BMT), also called as hematopoietic stem cell transplantation (HSCT). However, its application is limited by the cost and availability of few matched donors (7). Transfusion with RBC-rich blood is a useful and growing treatment option for controlling and avoiding SCD complications but this approach also has several limitations (4). The currently available United States Food and Drug Administration (US FDA)-approved therapeutic options for SCD patients include hydroxyurea (HU), L-glutamine oral powder (Endari^®^), intravenous Crizanlizumab (Adakveo), and Voxelotor/GBT440 (Oxbryta). Among these, HU and Voxelotor target hemoglobin S (HbS) polymerization whereas L-glutamine and Crizanlizumab target vaso-occlusion (4).

Oxidative stress is one of the key contributors to the pathophysiology of SCD and related complications (8, 9). It is reported that patients with SCD experience increased oxidative stress due to increased production of reactive oxygen species, mainly during vaso-occlusion and ACS (8). A previous *in vitro* study revealed that adhesion and damage of RBC membrane were affected by depletion of glutamine and patients with SCD have increased uptake of L-glutamine (9). L-glutamine, a precursor of nicotinamide adenine dinucleotide (NAD), may play an important role in the regulation of oxidative stress by normalizing the altered NAD redox system in patients with SCD (10). Indeed, L-glutamine increases the NADH and NAD redox potential to increase the amount of free glutamine in the blood. Sickle-shaped RBCs take up this free glutamine and use it to generate antioxidant molecules. These new antioxidants help to neutralize the oxidative stress in sickle cells (11). In the pivotal Phase 3 clinical trial, L-glutamine demonstrated significant reduction in pain crises, hospitalizations, and ACS events compared to placebo in patients with SCD with or without HU over a 48-week period (12). In September 2021, a re-analysis of the pivotal Phase 3 clinical data using a similar statistical method that was used for all approved medications (HU, Crizanlizumab, and Voxelotor) demonstrated that L-glutamine decreased the number of VOCs by 45% (13).

Endari^®^ is a pharmaceutical-grade L-glutamine approved by the US FDA in July 2017 for reducing acute complications of SCD in patients 5 years of age and older (14). After this announcement, Endari^®^ became the first approved treatment for children with SCD and the first innovative treatment for adults with SCD in almost two decades.

The objective of this study is to confirm the efficacy of L-glutamine therapy in both pediatric and adult patients with SCD at follow-up time points of 24, 48 and 72 weeks. Here, we present the preliminary results from the analysis of data from 19 patients who were initially enrolled of the120 patients who are planned to be recruited in this study.

## Materials and methods

### Study design

This observational study was conducted from October 2019 through April 2021 and included patients with confirmed SCD diagnosis with standard laboratory investigations obtained prior to initiation of L-glutamine therapy under the Early Access Programs (EAPs) approved by the local ethics committee (MRC-04-20-1240 in Qatar and Commission Nationale Informatique et Libertés approval Number 3Yj157849 3 in French Guiana). Written informed consent was obtained from patients or their parents or legal guardian. Patients were treated with L-glutamine and examined at follow-up time points of 24, 48 and 72 weeks.

### Treatment plan

Pharmaceutical-grade L-glutamine (Endari^®^; Emmaus Medical, Inc.) was administered orally twice daily for 72 weeks at a dose recommended in the package insert (0.3 g per kg of body weight per dose). Patients with severe VOC and ACS were hospitalized. Blood transfusion was given in case of ACS and uncontrolled pain associated with VOC despite administration of the highest dose of intravenous (IV) narcotic.

### Data collection

The following data were collected at:

(1) Baseline—age, gender, SCD genotype (HbSS), weight, age at the time of diagnosis, HU use and laboratory parameters including hemoglobin levels, hematocrit, WBC count, reticulocyte count, lactate dehydrogenase (LDH) levels. (2) The 12 months prior to therapy initiation (considered as baseline values)—clinical parameters including number of hospitalizations, days spent in hospital, VOCs, ACS events, and number of packed red blood cells (PRBCs) transfusions. (3) Follow-up clinic visits at 24, 48 and 72 weeks—both laboratory and clinical parameters. The data values for clinical parameters at 24, 48, and 72 weeks have been annualized. Severe VOC was defined as either ACS or painful crisis requiring IV narcotic analgesics.

### Safety outcomes

Any patient and/or healthcare practitioner-reported adverse events (AEs) or serious adverse events (SAEs) experienced during Endari^®^ treatment and follow-up clinic visits were recorded.

### Statistical analysis

It is planned to achieve a target sample size of 120 globally. For the clinical observations, non-parametric analysis was performed using the Friedman test with *P* < 0.05 for statistical significance followed by calculation of mean rank for multiple comparisons. The laboratory observations were analyzed with repeated measures ANOVA at *P* < 0.05 followed by *post-hoc* analysis with Bonferroni correction at *P* < 0.02 for significance. Statistical analysis was performed using MedCalc software Version 20.015 (MedCalc Software Ltd, Belgium).

## Results

### Baseline characteristics

A total of 19 patients (four patients from Qatar and fifteen patients from French Guiana) with confirmed diagnosis of SCD and having HbSS genotype were initially enrolled and retrospectively analyzed for this study. The 4 patients from Qatar had Arab-Indian haplotype and 15 patients from French Guiana had African haplotype of SCD. All 19 patients completed the study. The median age of patients was 17 years (range 8–54 years) with 53% of patients below 18 years and 47% above 18 years, and median weight was 50 kg (range 25-75 kg). More patients were males than females (53% vs. 47%). Higher proportion of patients received 15 g of L-glutamine twice daily (58%) than patients receiving L-glutamine at 10 g twice daily (42%). At baseline, 63% of the patients were taking HU therapy, but during the follow-up clinic visits only 47% had continued HU therapy in addition to L-glutamine (Table 1).

**Table 1:** Baseline characteristics of patients with sickle cell disease (SCD).

	**Patients with SCD (*****N*** = **19)**
Age, years	
Median	17
Range <18 years >18 years	8-54 10 (53%) 9 (47%)
Gender, *n* (%)	
Females	9 (47%)
Males	10 (53%)
SCD genotype	HbSS
Race	
Black	15 (79%)
Arab	4 (21%)
Weight, kg	
Median	50
Range	25-75
Age at time of SCD of diagnosis, *n* (%)	
At Birth	12 (63%)
Endari^®^ dose, g (twice daily), *n* (%)	
10 g	8 (42%)
15 g	11 (58%)
HU therapy at baseline, *n* (%)	
Yes	12 (63%)
No	7 (37%)
HU therapy at follow-up time points, *n* (%)	
Yes	9 (47%)
No	10 (53%)

### Clinical observations

Compared to baseline, patients had significantly fewer number of annual pain crises (VOCs) at 24, 48, and 72 weeks following L-glutamine therapy (median change from 3.0 to 0.0; *P* < 0.00001). The *post-hoc* analysis revealed a statistically significant difference at 72 weeks compared to baseline, 24 and 48 weeks (*P* < 0.05) (Table 2). The rate of annual hospitalizations was significantly reduced over 72 weeks from baseline (median change from 3.0 to 0.0; *P* < 0.00001). In addition, after treatment with L-glutamine, patients spent fewer days in hospital compared to baseline (median change from 15.0 to 0.0; *P* < 0.00001) (Table 2). The data at 72 weeks was found to be statistically significant in comparison to baseline and other two time points (*P* < 0.05). Compared to baseline, the number of blood transfusions were significantly lower at follow-up time points of 24, 48- and 72-weeks following L-glutamine therapy (median change from 3.0 to 0.0; *P* < 0.00001) (Table 2). In the year prior to therapy initiation, a total of 11 ACS events were reported in nine patients. However, after 48 weeks of L-glutamine therapy, only two such events were observed (Table 2). The general well-being of patients improved considerably with L-glutamine therapy without any treatment-related AEs (data not shown).

**Table 2:** Clinical observations of patients with sickle cell disease (SCD) at baseline and follow-up time points.

	**Follow–up time points**					
**Clinical endpoints**	**Baseline (*****N*** = **19)**	**24 weeks (*****N*** = **19)**	**48 weeks (*****N*** = **19)**	**72 weeks (*****N*** = **19)**	**P** ^*^	**P**
Annualized no. of VOCs						
Median (Range)	3(1–14)	0(0–6)	0(0–6)	0(0–6)	<0.00001	<0.05^†^
Total no. of ACS events (n)	11	NA	2	NA	NA	NA
Annualized no. of hospitalizations						
Median (Range)	3(1–8)	0(0–8)	0(0–6)	0(0–6)	<0.00001	<0.05^†^
Annualized no. of days spent in hospital						
Median (Range)	15(3–30)	0(0–24)	0(0–24)	0(0–16)	<0.00001	<0.05^§^
Annualized no. of blood transfusions						
Median (Range)	3(0–6)	0(0–4)	0(0–4)	0(0–0)	<0.00001	<0.05^§^

* Friedman test.

† Multiple comparisons: 72 weeks vs. baseline, 24 and 48 weeks.

§ Multiple comparisons: baseline vs. 24, 48 and 72 weeks.

NA, Not available.

Taken together, in this group of SCD patients, there was a significant decline in the median (Table 2) as well as average (Supplementary Table 1) number of annual VOCs, hospitalizations, days spent in the hospital, and blood transfusions over 72 weeks from baseline following L-glutamine therapy.

### Laboratory observations

**Figure 1:**
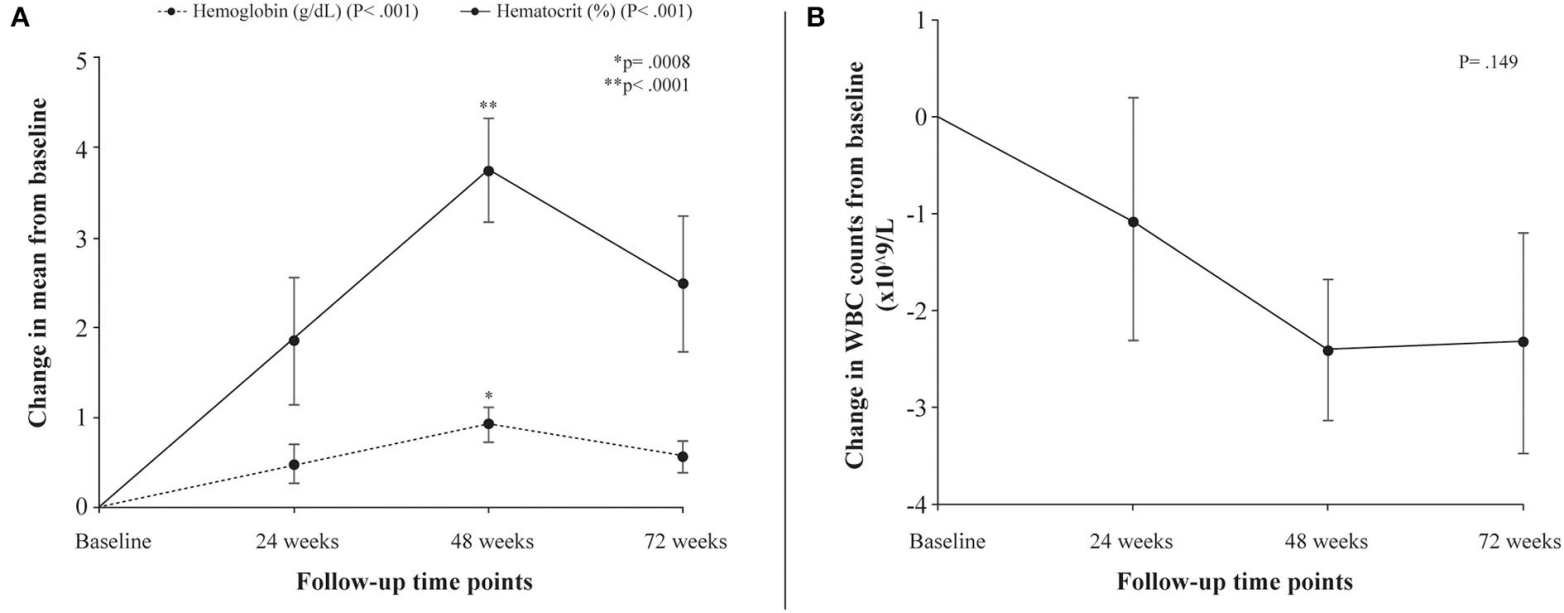
Differences in laboratory parameters from baseline to follow-up time points. **(A)** Change in mean levels of hemoglobin (g/dL) and hematocrit (%) from baseline to follow-up time points. **(B)** Change in mean WBC counts from baseline to follow-up time points. P denotes probability values for repeated measures ANOVA; p denotes probability value of pairwise comparison of mean difference (Bonferroni correction) in hemoglobin levels (^*^) and hematocrit (^**^) at 48 weeks from baseline.

**Figure 2:**
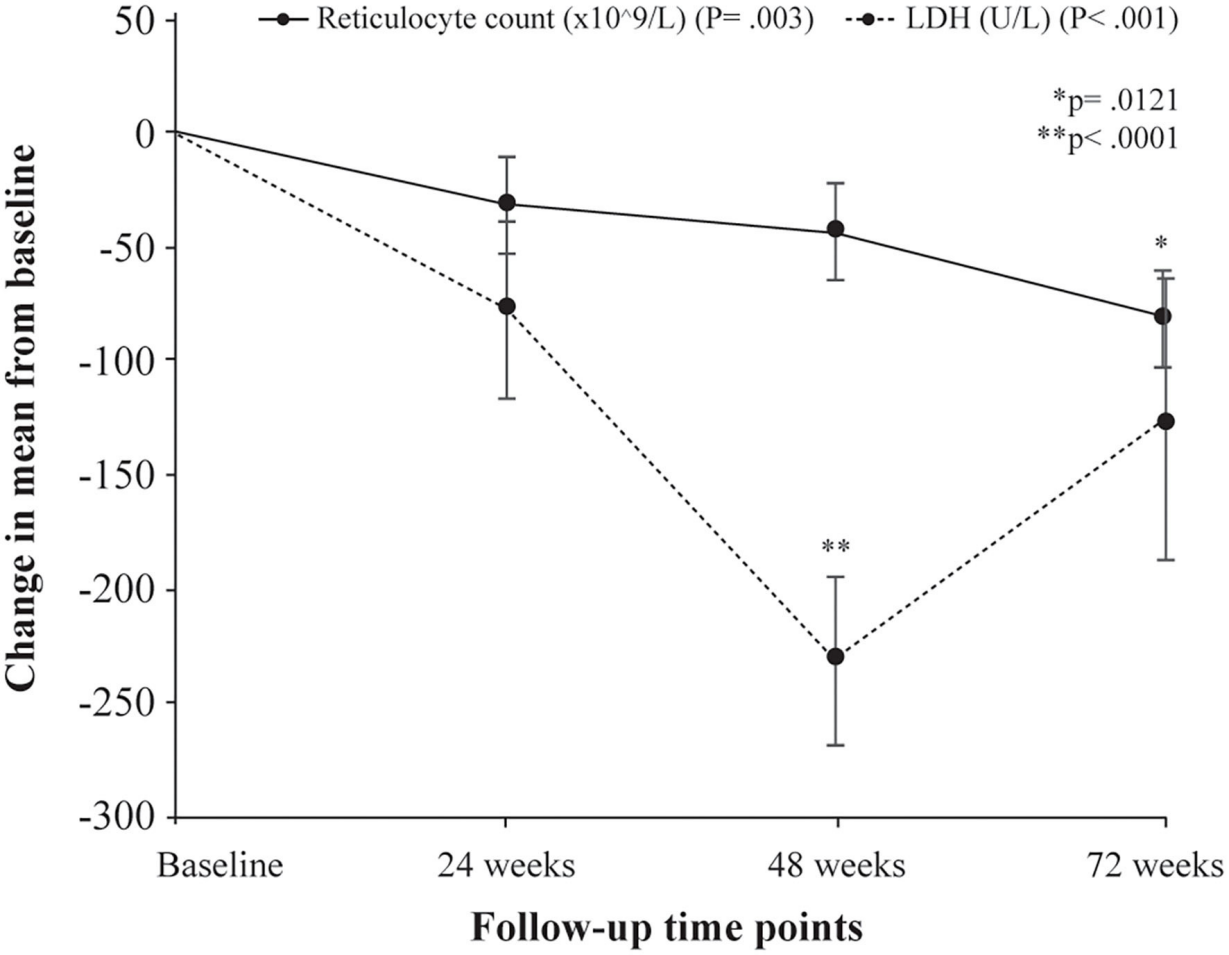
Change in mean levels of hemolysis markers (reticulocyte counts and LDH levels) from baseline to follow-up time points. P denotes probability values for repeated measures ANOVA; p denotes probability value of pairwise comparison of mean difference (Bonferroni correction) in reticulocyte counts at 72 weeks from baseline (^*^) and LDH levels at 48 weeks from baseline (^**^).

Following treatment with L-glutamine, the mean levels of hemoglobin increased significantly from baseline to 72 weeks (8.2 to 8.8 g/dL; *P* < 0.001) with peak mean increase from baseline of 11.2% at 48 weeks. The *post-hoc* analysis showed mean difference in hemoglobin levels from baseline to be statistically significant at 48 weeks (*P* = 0.0008). The mean hematocrit proportions increased markedly from baseline to 72 weeks (24% to 27%; *P* < 0.001) with highest mean improvement from baseline of 15.5% at 48 weeks. The mean difference from baseline was found to be significant at 48 weeks (*P* < 0.0001) (Table 3, Figure 1A). There was a decrease from baseline in the average WBC counts at the follow-up time points but the difference was not statistically significant (*P* = 0.149) (Table 3, Figure 1B). A similar pattern of decline in mean reticulocyte counts from baseline was observed at 24, 48 and 72 weeks (284.4 to 203.6 x10^9^/L; *P* = 0.003) with mean difference from baseline reaching statistical significance at 72 weeks (*P* = 0.0121) (Table 3, Figure 2). The mean LDH levels significantly decreased at the follow-up time points compared to baseline (561.8 to 436.4 U/L; *P* < 0.001) and mean difference from baseline was found to be significant at 48 weeks (*P* < 0.0001) (Table 3, Figure 2).

**Table 3:** Laboratory parameters of patients with sickle cell disease (SCD) at baseline and follow–up time points.

	**Follow–up time points**								
**Laboratory parameters**	**Baseline (*****N*** = **19) Mean** ±**SE**	**24 weeks (*****N*** **=** **19) Mean** ±**SE**	**Change in mean from baseline to 24 weeks**	**48 weeks (*****N*** = **19) Mean** ±**SE**	**Change in mean from baseline to 48 weeks**	**72 weeks (*****N*** = **19) Mean** ±**SE**	**Change in mean from baseline to 72 weeks**	**P** ^*^	**P**
Hemoglobin, g/dL	8.2 ± 0.35	8.7 ± 0.27	0.48 ± 0.20	9.2 ± 0.26	0.92 ± 0.19	8.8 ± 0.33	0.57 ± 0.18	<0.001	0.0008^†^
Hematocrit, %	24.1 ± 0.92	26.0 ± 0.86	1.86 ± 0.71	27.9 ± 0.83	3.75 ± 0.58	26.6 ± 1.10	2.49 ± 0.76	<0.001	<0.0001^†^
WBC count, x10^9^/L	11.9 ± 0.97	10.8 ± 1.10	−1.0737 ± 1.24	9.5 ± 0.83	−2.41 ± 0.71	9.6 ± 0.83	−2.32 ± 1.14	0.149	0.0205^†^
Reticulocyte count, x10^9^/L	284.4 ± 21.72	253.0 ± 19.18	−31.37 ± 21.45	241.6 ± 17.44	−42.8 ± 20.61	203.6 ± 19.85	−80.74 ± 22.39	0.003	0.0121^‡^
LDH, U/L	561.8 ± 50.68	484.9 ± 43.37	−76.90 ± 38.72	331.2 ± 26.85	−230.63 ± 37.26	436.4 ± 48.95	−125.42 ± 61.89	<0.001	<0.0001^†^

SE, Standard error.

* Repeated measures ANOVA.

Pairwise comparison of mean difference (Bonferroni correction):

† baseline vs. 48 weeks;

‡ baseline vs. 72 weeks.

## Discussion

This study including 19 patients with SCD and nearly half of the patients receiving HU simultaneously (47%) demonstrated a clinically significant reduction in the median frequency of VOCs at 24, 48, and 72 weeks following L-glutamine therapy compared to the preceding year. Compared to baseline, the number and duration of hospitalizations were significantly reduced at all follow-up time points. These results are consistent with published data which indicates that about 95% of patients with SCD get hospitalized due to severe episodes of pain or VOCs (15) and alleviating VOCs reduces both the frequency and duration of hospitalizations (16). ACS is one of the common reasons for hospitalization and a topmost cause of mortality among patients with SCD (17). In this study, L-glutamine therapy resulted in considerably lesser number of ACS events in patients at the follow-up time points.

Following L-glutamine therapy, significant changes were observed in the hemolysis parameters. Compared to baseline, both hemoglobin and hematocrit increased in patients at 24, 48, and 72 weeks from the start of L-glutamine therapy with peak mean increase from baseline of 11.2% and 15.5% respectively at 48 weeks. The median number of blood transfusions were significantly reduced from 15 in the preceding year to none following treatment with L-glutamine. This would result in fewer transfusion-related adverse events (AEs) and complications (4). Several clinical studies have demonstrated elevated levels of serum LDH in sickle cell patients in steady state due to oxidative stress associated with hemolysis (18, 19). For this reason, LDH is used as a marker of hemolysis in SCD (20). The LDH levels are significantly much higher during painful crises (21). In our study, the serum levels of LDH decreased dramatically from baseline at all follow-up time points following L-glutamine therapy (561.8 to 436.4 U/L). This suggested a decrease in hemolysis with an associated reduction in VOC. It is important to note here that improved hemolysis parameters do not always indicate reduced VOCs. As an example, Voxelotor showed improvement in hemolysis with no reduction in VOCs (22), whereas Crizanlizumab showed decreased VOCs without improvement of hemolysis parameters (23).

It is reported that sickle cell patients have higher number of reticulocytes compared to non-sickle cell subjects (24). The increased reticulocytosis is caused by chronic peripheral hemolysis (25). This supports the use of elevated reticulocyte count as a marker of hemolysis in SCD (20). Our results showed significant reduction in reticulocyte counts from baseline through 72 weeks after treatment with L-glutamine. Leukocytes or WBCs contribute to the pathophysiology of SCD (26). In a cohort study, the number of WBCs was higher in SCD patients compared to non-SCD subjects (24). However, reducing the number of leukocytes ameliorates SCD (26). Supporting this, a double-blind randomized clinical study showed the association of decrease in neutrophil count with clinical efficacy of hydroxyurea in SCD patients (27). Consistent with these observations, in the present study, treatment with L-glutamine resulted in mean decrease in WBC counts of up to 20% from baseline to 72 weeks.

There were no L-glutamine-associated AEs reported by patients or healthcare practitioners, thus confirming the reported safety profile (12). Thus, L-glutamine (Endari^®^) provides beneficial effects in patients unable to receive hydroxyurea or who may have undesirable side effects from hydroxyurea or in addition to hydroxyurea to lower the incidence of pain crises for those who may have partial response to HU (28).

This study provides the description of real-world data on L-glutamine therapy in patients with SCD supporting the previously published results (12). Most importantly, for the first time, the clinical and laboratory findings from this study demonstrate significant improvement in clinical parameters along with improvement of hemolysis parameters. These promising results may have influenced patients' willingness to continue L-glutamine medication. The small sample size is the main limitation of this study; however, with the anticipated approval of L-glutamine (Endari^®^) in Qatar, larger studies will be conducted to confirm these encouraging results.

## Conclusion

This study demonstrated for the first time that L-glutamine (Endari^®^) therapy in SCD patients from Qatar and French Guiana resulted in significant improvements in clinical outcomes (number of VOCs, number and duration of hospitalizations, and number of blood transfusions) accompanied by noteworthy increases in hemoglobin levels and reductions in markers of hemolysis (reticulocyte counts and LDH levels). Moreover, there was a drastic decrease in the number of ACS events following L-glutamine therapy. Furthermore, treatment with L-glutamine resulted in clinically significant outcomes from baseline through 72 weeks suggesting sustained long-term efficacy.

## Data availability statement

The raw data supporting the conclusions of this article will be made available by the authors, without undue reservation.

## Ethics statement

The studies involving human participants were reviewed and approved by Local Ethics Committee (Hamad Medical Corporation - PO Box 3050, Doha - Qatar, MRC-04-20-1240 in Qatar and Commission Nationale Informatique et Libertés - 3 Place de Fontenoy - 75007 Paris, France, approval number 3Yj157849 3 in French Guiana). Written informed consent to participate in this study was provided by the participants' legal guardian/next of kin.

## Author contributions

NE and MY: conceptualization, investigation, methodology, validation, and writing—review and editing. GL, ME-J, RA-O, and AA: validation and writing—review and editing. All authors contributed to the article and approved the submitted version.

## Conflict of interest

NE and MY had received fees for consultancy from Emmaus Medical, Inc. The remaining authors declare that the research was conducted in the absence of any commercial or financial relationships that could be construed as a potential conflict of interest.

## Publisher's note

All claims expressed in this article are solely those of the authors and do not necessarily represent those of their affiliated organizations, or those of the publisher, the editors and the reviewers. Any product that may be evaluated in this article, or claim that may be made by its manufacturer, is not guaranteed or endorsed by the publisher.
