# Supplementary material for: Real-World data on efficacy of L-glutamine in preventing sickle cell disease-related complications in pediatric and adult patients
Source: Front Med (Lausanne). 2022 Aug 1;9:931925. doi: 10.3389/fmed.2022.931925 (PMC9376442; doi:10.3389/fmed.2022.931925)
Supplement: Supplementary file 1 [file Table_1.DOCX]

**Supplementary Table 1**. Change in clinical parameters of patients with sickle cell disease (SCD) from baseline to 72 weeks

|  |  | |  | | **Follow-up time points** | | | | |  |  |  | |
| --- | --- | --- | --- | --- | --- | --- | --- | --- | --- | --- | --- | --- | --- |
| **Clinical Endpoints** | | **Baseline**  **(N=19)**  **mean ±SE** | | **24 weeks**  **(N=19)**  **mean ±SE** | | **% change in mean from baseline to 24 weeks** | **48 weeks**  **(N=19)**  **mean ±SE** | **% change in mean from baseline to 48 weeks** | **72 weeks**  **(N=19)**  **mean ±SE** | **% change in mean from baseline to 72 weeks** |  | **P value*** |  |
| Annualized no. of VOCs | | 4.32±0.93 | | 1.58±0.62 | | -63.4 | 1.26±0.53 | -70.7 | 0.53±0.34 | -87.8 |  | <0.00001 |  |
|  | |  | |  | |  |  |  |  |  |  |  |  |
| Annualized no. of hospitalizations | | 3.26±0.43 | | 1.47±0.59 | | -54.8 | 1.47±0.55 | -54.8 | 0.53±0.34 | -83.9 |  | <0.00001 |  |
| Annualized no. of days spent in hospital | | 13.16±1.78 | | 4.42±1.67 | | -66.4 | 5.79±2.12 | -56.0 | 1.16±0.86 | -91.2 |  | <0.00001 |  |
|  | |  | |  | |  |  |  |  |  |  |  |  |
| Annualized no. of blood transfusions | | 3.06±0.57 | | 0.33±0.24 | | -89.1 | 0.22±0.22 | -92.7 | 0.0±0.0 | -100.0 |  | <0.00001 |  |

SE: Standard error

*Repeated measures ANOVA
